# Predictors of unmet needs among people with diabetes mellitus type 2 in Gampaha district of Sri Lanka

**DOI:** 10.1371/journal.pgph.0002462

**Published:** 2024-10-10

**Authors:** Nimali Widanapathirana, Rajitha Wickremasinghe, Susie Perera, Martin McKee, Benjamin Palafox, Dina Balabanova

**Affiliations:** 1 Ministry of Health, Colombo, Sri Lanka; 2 Faculty of Medicine, University of Kelaniya, Ragama, Sri Lanka; 3 Faculty of Public Health and Policy, London School of Hygiene and Tropical Medicine, London, United Kingdom; University of South Carolina Arnold School of Public Health, UNITED STATES OF AMERICA

## Abstract

Diabetes mellitus is a significant contributor to the disease burden in Sri Lanka. Despite government efforts to improve access to care for those with chronic illness, major gaps remain. We assessed the prevalence and correlates of unmet needs among persons with diabetes mellitus type 2 to inform policies on improving healthcare access in a predominantly tax-funded public healthcare system. A descriptive cross-sectional study identified 401 persons with diabetes mellitus type 2 using a multi-stage cluster sampling method from 1767 individuals aged 40–69 from the Gampaha district, just north of the capital Colombo. An interviewer-administered questionnaire gathered data on unmet needs for physician care, medicines, and investigations during the preceding year. Associated factors, identified from the health behaviour model, were examined using binomial logistic regression with significance set at p<0.05. One-fifth experienced an unmet need (95%CI:15.7–23.7), with 16% for physician care (95%CI:12.7–20.2), 4.2% for medicines (95%CI:2.5–6.7) and 6.0% for investigations (95%CI:3.9–8.8). People who frequently visited a private provider experienced less unmet needs overall. Being female (AOR 0.50; 95%CI:0.27–0.92) and having a higher income (AOR 0.37; 95%CI:0.16–0.83) reduced unmet need for physician care. Absence of other major chronic illnesses (AOR 0.31; 95%CI:0.12–0.80) and having a regular care provider in the public (AOR 0.24; 95%CI:0.07–0.89) or private sectors (AOR 0.18; 95%CI:0.05–0.68) reduced unmet need for investigations with the latter also reducing unmet need for medicines (AOR 0.11; 95%CI:0.02–0.77). Despite Sri Lanka having a predominantly publicly financed healthcare system, persons with diabetes mellitus experienced unmet healthcare needs, mainly for physician care which varied with socio-economic characteristics. It is important to ensure uninterrupted care, universally for all, through patient-centred models of care linked to a regular provider. Health planners should take account of unmet needs when expanding public sector coverage for chronic illness care.

## Introduction

In 2021, 537 million people aged 20–79 lived with diabetes mellitus type 2 globally, a number predicted to increase to 783 million by 2045 [1]. The hallmark of diabetes management is optimal glycaemic control. Despite a plethora of evidence-based effective interventions to achieve glycaemic targets, this often fails to translate into practice, leaving many who could benefit unable to access them. Most developed countries have transitioned from episodic management in acute care settings to regular people-centred care coordinated in primary care settings [2]. This transition is still a work in progress in many low- and middle-income countries, despite the high disease burden [3].

Sri Lanka, a lower middle-income country in Asia, was one of the countries singled out in the seminal Rockefeller Foundation report for achieving good health at a low cost with a tax-funded public healthcare system that provides services free at the point of delivery [4]. However, its health system has primarily evolved to respond to communicable diseases, characterized by episodic delivery of care. In this field, it has made impressive progress eliminating important communicable diseases such as polio, malaria, filariasis, and measles. However, it has struggled in the face of the epidemiological transition, characterised by an ageing population and an increasing number of people living with non-communicable diseases [5].

Diabetes mellitus is a significant contributor to the burden of non-communicable diseases in Sri Lanka, with a prevalence similar to that in high-income countries and higher than in many other middle-income countries [6]. While recent data are lacking, older studies report an increase in the prevalence from 2.5% in 1990 within a rural community to 10.3% in 2005 based on a nationally representative sample of adults over 18 years [7,8]. The 2021 International Diabetes Federation (IDF) Diabetes Atlas reports a crude prevalence of 9.8% (95% Confidence Interval (CI) 7.7–13.1) and an age-standardised prevalence of 11.3% (95% CI 9.1–14.5). Further, Sri Lanka ranks third among IDF Southeast Asian countries with respect to the age-adjusted prevalence and number of people with diabetes mellitus in the 20–79 year age group [9].

The health and survival of those with diabetes mellitus depend on a well-functioning health system [3]. Sri Lanka has a pluralistic health service delivery model where ambulatory care is provided in an approximately equal mix of private and public facilities, while most inpatient care is provided by government hospitals, which should, at least in theory, achieve this [6]. Diabetes services in Sri Lanka are provided in both the public and private sectors, as with other chronic diseases. Within the public sector, dedicated diabetes clinics operate at all levels of care, including primary, secondary, and tertiary levels, with the latter two providing specialist care [10]. Due to the absence of a gatekeeping mechanism at the primary level, individuals can seek care directly from a primary hospital or a higher-level hospital in the public sector [5]. Individuals have a right to choose where they will receive care for their diabetes within the public sector although, obviously, this is constrained by geography, levels of provision, and perceived or actual quality. Private provision, for which the patient must pay, is primarily carried out by doctors employed in the public sector who engage in dual practice, while private hospitals also play a role, particularly in urban areas [11]. The demand for private care is mostly driven by non-clinical aspects of care, such as interpersonal quality, longer consultation time, better communication with physicians, continuity of care, and the freedom to choose one’s physician [12].

Despite what appears to be a high level of provision, the Sri Lankan population still faces many challenges in accessing necessary healthcare, and significant unmet needs continue to be reported. While much of the available evidence pertains to the hospital sector, individuals with diabetes mellitus encounter numerous barriers in obtaining care [13]. Health expenditure data indicate that individuals with diabetes mellitus incur significant costs for medicines and ambulatory care [6]. They bypass the free public services to seek private care or purchase medicines from private pharmacies when those provided free at public health facilities are unavailable [14].

Addressing these unmet needs is now a priority for the government. A range of initiatives has been adopted, including the implementation of an essential services package based on “shared care clusters”. These clusters bring together facilities within a defined geographical area to provide care close to people’s homes, with family doctors playing a key role [15,16]. Guidelines for chronic disease management and a corresponding essential drugs list have also been introduced to streamline service delivery [17].

8Unmet healthcare needs for diabetes mellitus indicate the presence of barriers to access, often affecting different groups within populations differentially [18]. A 2014 STEP survey found that approximately 30% of those aged 18–69 with a self-reported history of elevated blood sugar were not taking any medication [19]. However, the published report only provided disaggregated data by age group and sex, without reporting any differences based on socioeconomic characteristics.

This study aims to measure the extent of unmet health needs among individuals with diabetes mellitus type 2 in a district in Sri Lanka. Unmet need is defined as the foregone care during the preceding 12 months. Additionally, we examined how unmet health needs varied across a range of socioeconomic characteristics.

## Methods and materials

### Study design and setting

We conducted a population-based survey among persons aged 40–69 years in the Gampaha district of Sri Lanka. This was a comprehensive survey that examined the effective coverage of diabetes mellitus type 2 in this population. As part of this survey, we assessed the unmet healthcare needs among individuals with diabetes mellitus that we report in this paper.

Gampaha district is the second most populous district in the country. It is located north of Colombo, with an estimated population of 2,304,833, comprising 11% of the total population of Sri Lanka [20]. Administratively, the Gampaha district is divided into 13 Divisional Secretariat (DS) divisions, with a total of 1,177 Grama Niladhari (GN) divisions. Divisional Secretariats are the third level of administrative division in the country, while GN divisions are the lowest level. The first two levels of administrative division are the provinces and districts. Gampaha district is one of the three districts in the Western province.

Furthermore, the local government system of Sri Lanka consists of three tiers of legislative bodies: Municipal Councils, Urban Councils, and Pradeshiya Sabhas. In the Gampaha district, there are two Municipal Councils, five Urban Councils, and 12 Pradeshiya Sabhas, each comprising several GN divisions. The Municipal Councils and Urban Councils are officially recognized as “urban areas”, while the Pradeshiya Sabhas are classified as “rural areas”. Based on this classification, the majority of the population in the Gampaha district resides in rural areas, accounting for 84.3% [20].

The public sector in the Gampaha district provides health care through three tertiary care hospitals, three secondary care hospitals and 57 primary care hospitals dispersed within the district [21]. However, Sri Lanka does not collect systematic data on private sector providers.

#### Survey design

We used a measure of glycaemic control, taken from a survey conducted in 2012, to calculate the sample size for the survey. The 2012 survey indicated that 84.6% individuals with diabetes mellitus were not optimally controlled as measured by HbA1c [22]. It has been used as a measure of effective coverage in the first global monitoring report on tracking universal health coverage [23]. We considered this as a proxy measure of unmet healthcare needs, although not exactly what we were assessing: self-reported unmet need for care (in terms of access to a health worker, to medicines, and to investigations) among people with diabetes mellitus type 2. However, there was no previous survey in Sri Lanka that would provide an initial estimate of self-reported unmet need for care.

We used the proportion of people without optimal control of diabetes mellitus to calculate the sample size.

The study applied the sample size computational formula which considered estimates of a proportion [24] with a 5% margin of error and 95% confidence level of the standard normal distribution at Z = 1.96. We got a sample size of 196.

The next step was to estimate the number of subjects needed to identify 196 individuals with type 2 diabetes. We found four recent studies that reported prevalence, albeit in different population groups. Wijewardene et al. conducted a survey among individuals aged 30–65 years in four provinces, reporting a prevalence of 14.2% (CI 11.9–16.5) in men and 13.5% (CI 6.9–20.1) in women [25]. A nationally representative survey reported a prevalence of 10.3% (9.4–11.2%) in those aged 20 and over [26]. Another study, conducted in a single district, reported a prevalence of 14.7% among individuals aged 35–64 years [27] while a fourth study reported figures of 20.3% in men and 19.8% in women aged 35–64 years [22]. Since the last study closely aligned with the age group of interest and was conducted in the same region, we adopted a prevalence estimate of 20%. Multiplying our target value up, this yielded a figure of 980. However, this calculation did not account for the cluster design we planned to use. To address this, we applied the following formula:

N=nxD

D = 1+(b-1),where b is the number of households in a cluster and ρ is the intra-class correlation coefficient [28].

We pre-selected a cluster size of 30, based on our knowledge of settlements in the district. With this figure the design effect can take a value of 0.6 to 6. Taking a conservative estimate, we assigned a design effect as 2, effectively doubling the required sample size. We then allowed for a 10% non-response rate, yielding a final sample size of 2177. To accommodate clusters of 30 households, we determined that 73 clusters would be needed, which we rounded up to 75.

A multi-stage cluster sampling method was used. The primary sampling units were the Grama Niladhari (GN) divisions. The number of residents aged 40 to 69 years in each GN division was obtained from the Department of Census and Statistics [29]. The sampling frame consisted of all GN divisions in Gampaha, and 75 divisions were selected proportionally based on population size. Then, using the electoral list of a GN division as the sampling frame, an initial household was selected at random from each GN division. Starting from this household, the next 29 houses were chosen in a pre-determined manner. The fifth household moving in a left-handed direction from the front door was selected until all 30 households in the cluster were identified. Eligible participants were adults aged 40 to 69 years who had lived in the area for at least six months. Those seriously ill, pregnant, or lactating at the time of the survey were excluded. If there was no answer after 3 visits or the household contained no eligible subject, the next one was chosen. From each selected household, one individual was randomly chosen. Those who refused to participate were recorded as non-responders. The study achieved a response rate of 78.5%, resulting in 1,766 participating individuals. Among them, 401 (22.7%) already had a diagnosis of type 2 diabetes, exceeding our sample size calculation. We verified the diagnosis by examining the medical records provided by the respondents and assessing their current treatment.

#### Assessment of unmet need for diabetes care

Unmet healthcare needs are defined as the gap between services deemed necessary to adequately address health problems and the health services received [30]. Self-reported unmet need for healthcare is used in many international surveys to quantify the level of unmet needs for different disease states [31,32]. Typically, this is assessed by asking individuals whether they have foregone care or did not receive the necessary care within a specified period [18].

When access to health services is not realized it manifests as an unmet need [33]. The questions were developed following a review of relevant literature [32–36]. This review suggested the inclusion of three questions: “In the last 12 months, how many times were you unable to meet your diabetes care provider even when you wanted to?”; “In the last 12 months, how many times were you unable to get medicine/insulin prescribed by your diabetes care provider?”; and “In the last 12 months, were you unable to get the investigations prescribed by your diabetes care provider for managing your diabetes?”. The latter referred to investigations prescribed by the diabetes care provider including investigations such as fasting blood glucose and HbA1C.

Unmet needs can arise due to barriers associated with the healthcare system and personal characteristics of patients [18,37]. Respondents who replied ‘yes’ to any of the above questions were further asked to select the reason for the unmet needs from a list of barriers to accessing care that was identified from the literature review (S1 Text). We classified the responses based on the concept of access described by Penchansky and Thomas [38], incorporating methods adopted by Chen and Hou [37], and Ronksley et al. [39]. These categories were: availability (perceived deficiencies in the volume, type, and manner of service delivery that impact healthcare utilization), accessibility (barriers that restrict or impede the use of available healthcare services), and acceptability (perceptions on provider characteristics), and personal choice (personal attitudes and circumstances that impact healthcare use unrelated to the healthcare system).

#### Determinants of unmet need

**Fig 1:**
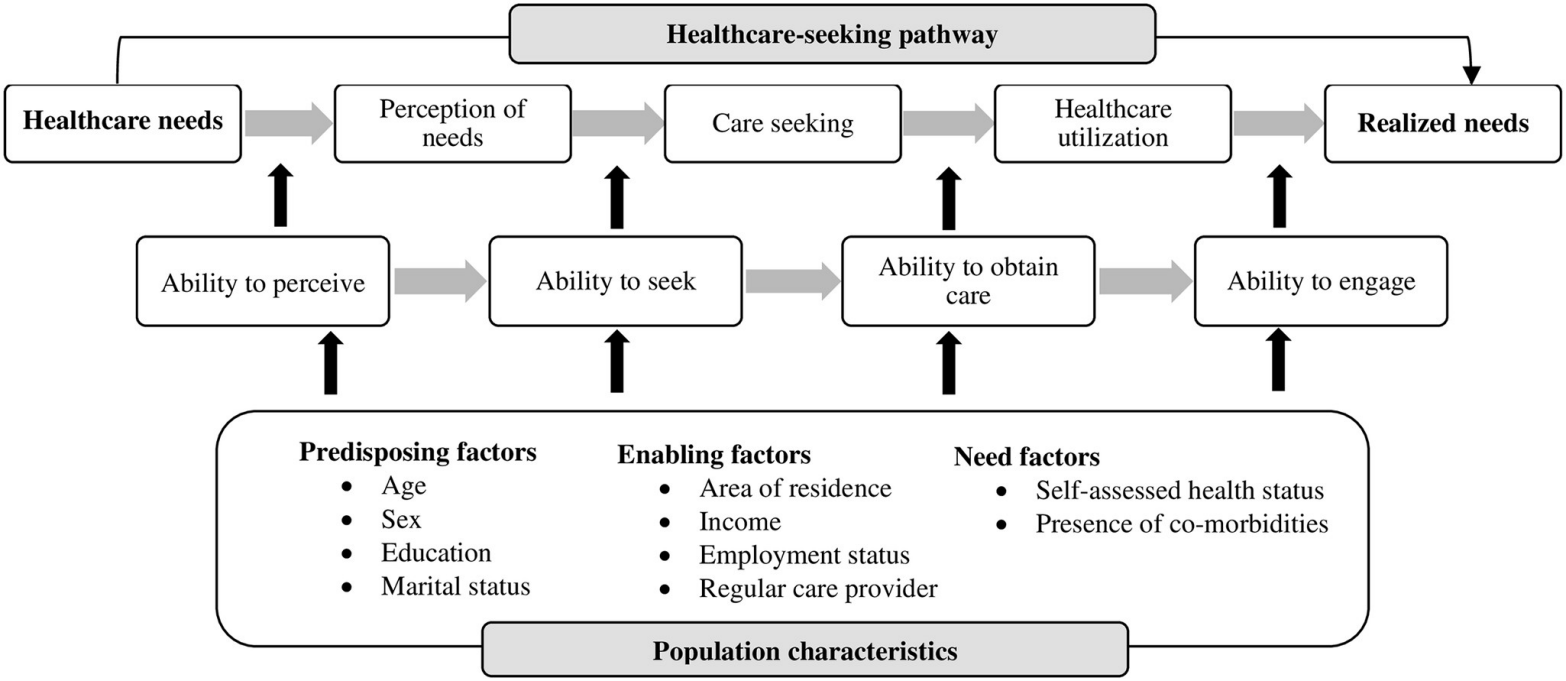
Framework on unmet healthcare needs. Adapted from Aday and Andersen [40], and Levesque, Harris, and Russel [41].

The next step was to assess the determinants of unmet needs. We developed a conceptual framework by adapting the health behaviour model described by Aday and Andersen [40] and the concept of access modelled by Levesque, Harris, and Russel [41]. The health behaviour model has been widely used in health services research [42]. It characterizes patients in terms of predisposing, enabling, and need factors that either facilitate or impede their interactions with the healthcare system. Predisposing factors reflect the individual’s propensity to use health services, such as age, sex, level of education and marital status. Enabling factors refer to resources that may facilitate access to services, such as household income, employment status, area of residence, and having a regular care provider. Need factors represent a person’s immediate reason for using healthcare, which may be perceived by the individual or determined by a healthcare professional, such as perceived health status or the presence of co-morbidities [40,42]. Levesque, Harris, and Russel [41] present a synthesis of the concepts of access and introduce five abilities of patients and five dimensions of accessibility that interact to generate access. We amalgamated the main features of the models on healthcare access described by these authors and developed a framework (Fig 1) to understand the determinants of unmet needs based on the predisposing, enabling and need factors.

We gathered data on predisposing factors, which included the age, sex, marital status, and education level of respondents. Need related factors comprised the perceived health status and the presence of other major non-communicable diseases such as cardiovascular disease, cerebrovascular disease, and chronic respiratory disease. The enabling factors included the income level, area of residence, employment status and usual source of care for diabetes mellitus. The definitions of independent variables measured in the study are given in Table 1.

**Table 1:** Definitions of independent variables.

Independent variable	Description
**Predisposing factors**	
Age	Categorised as 60 and above and below 60 years, 60 being the age of retirement in Sri Lanka in the public sector
Sex	Male or Female
Education	Classified as educational achievement up to Grade 10 and above Grade 10
Marital status	Categorized as ‘yes’ if having a partner at present and ‘no’ if not having a partner at present
**Enabling factors**	
Area of residence	Urban or rural based on the status of the Grama Niladhari division
Household income	Defined by income quintiles according to the national household income and expenditure survey [43] and classified into two categories such that income groups 1,2 and 3 were categorised as low-income and income categories 4 and 5 as high-income, and people who did not declare the level of income as undeclared
Employment status	Categorized as ‘yes’ if currently employed or ‘no’ if not employed
Regular care provider	Defined as the healthcare provider the respondents considered as their principal provider for diabetes care and regularly sought care from for diabetes. Three categories: not having a regular care provider, having a regular care provider in the government sector, and having a regular care provider in the private sector
**Need factors**	
Self-assessed health status	Two categories, those who responded either “very good” or “good” classified as “good” and the others as “bad”
Presence of another chronic condition	Categorized as ‘yes’ and ‘no’ based on the answer to the question on the presence of any of four major non-communicable diseases: chronic respiratory illnesses, cancer, cerebrovascular diseases, and cardiovascular diseases

#### Data collection tool

An interviewer-administered questionnaire was deemed the most appropriate method for this study, considering the diverse education levels of the study participants. The questionnaire was pre-tested among individuals meeting the eligibility criteria selected from Grama Niladari divisions from the Gampaha district that were not included in the final sample. The selection process utilized a convenience sampling technique.

#### Data analysis

The overall prevalence of unmet need was determined by aggregating responses to the questions on unmet need for physician care, medicines, and investigations. We calculated the prevalence of unmet needs based on the type of provider accessed. The differences between the groups were evaluated using χ2 tests. Additionally, we conducted three separate binomial logistic regression analyses to examine the determinants of unmet need for physician care, medicines, and investigations. The dependent variable for each analysis was the presence of unmet need for the respective type of care. The independent variables included the predisposing, enabling and need factors described in Table 1, and the models were adjusted for all covariates in the table. Adjusted odds ratios with corresponding p-values were estimated. Furthermore, we reported socio-economic differences in the prevalence of unmet needs, considering factors such as age, sex, area of residence, education level, employment status, and annual family income. Statistical analyses were performed using SPSS version 23 (IBM Corp. Released 2015. IBM SPSS Statistics for Windows, Version 23.0. Armonk, NY: IBM Corp.) with a significance level set at p<0.05.

#### Ethics approval

The study was approved by the Ethics Review Committee of the Faculty of Medicine, University of Colombo, Sri Lanka (EC 15–127). Each study participant gave written informed consent for participation, and their privacy and confidentiality were ensured by maintaining anonymity. The local administrators were informed of the nature of the study, and their full cooperation obtained to conduct the study.

## Results

We included 401 individuals with known diabetes mellitus type 2, comprising 22.7% of the overall sample. Most were female (n = 232, 57.8%), aged 60 years or less (n = 234, 58.4%), educated up to Ordinary Level or above (n = 251, 62.6%), and unemployed (n = 254, 63.3%). Of the 350 who declared their income the majority were in lower income groups (n = 257, 73.4%). Nearly 10% lacked a regular diabetes care provider (n = 39), while 48% (n = 192) and 42% (n = 170) regularly sought care from the public sector and the private sector, respectively. During the preceding 12 months, almost equal proportions of persons with diabetes mellitus most frequently visited a diabetes care provider in the public sector (n = 203, 50.6%) or the private sector (n = 198, 49.4%).

Overall, 19.5% (n = 78, 95%CI: 15.7–23.7) of the sample reported an unmet need which was 16.2% (n = 65, 95%CI: 12.7–20.2) for physician care, 4.2% (n = 17, 95%CI: 2.5–6.7) for medicines and 6.0% (n = 24, 95%CI: 3.9–8.8) for investigations. An unmet need for either physician care, medicines or investigations was reported by 14.2% (n = 57, 95%CI: 10.9–18.0), for any two of these categories by 3.5% (n = 14, 95%CI: 1.9–5.8), and for all three categories: physician care, medicines and investigations, by 1.7% (n = 7, 95% CI: 0.7–3.6%). Table 2 reports unmet needs by type of healthcare provider most frequently accessed by patients in the preceding 12 months.

**Table 2:** Prevalence of unmet needs by type of provider most frequently visited by persons with diabetes mellitus type 2.

Type of unmet need	Public sector (n = 203)		Private sector (n = 198)		χ^2^ test*	p-value
	n	%	n	%		
Physician care	42	20.7	23	11.6	6.076	0.014
Medicines	14	6.9	3	1.5	7.150	0.007
Investigations	15	7.4	9	4.5	1.441	0.230
Any unmet need	50	24.6	28	14.1	7.039	0.008

*df = 1.

Unmet needs for physician care and medicines were higher among patients who sought care from the public sector, and they reported more unmet needs overall.

Table 3 summarises the top three reasons cited by the respondents for the reported unmet needs by availability, accessibility, and personal choice. Acceptability of services did not account for any unmet needs and thus was not included in the table.

**Table 3:** Frequency of reasons cited by respondents reporting an unmet need.

Reason for unmet need	Unmet need for physician care (n = 65)		Unmet need for medicines (n = 17)		Unmet need for investigations (n = 24)	
	n	(%)	n	(%)	n	(%)
**Availability**						
Not available during a feasible time	37	56.9	10	58.8	17	70.8
Long waiting time	10	15.4	-	-	-	-
Not available on the day of visit	3	4.6	1	5.9	8	33.3
**Accessibility**						
Lack of transport	21	32.3	1	5.9	6	25.0
Lack of financial resources	18	27.7	6	35.3	21	87.5
**Personal choice**						
Deemed not necessary	8	12.3	2	11.8	8	33.3
Personal responsibilities	8	12.3	5	29.4	5	20.8
No one to accompany	5	7.7	-	-	7	29.2

The majority could not seek physician care (n = 37, 56.9%) or get medicines (n = 10, 58.8%) due to services not being available at a time feasible for them to access. Lack of transport (n = 21, 32.3%) and lack of financial resources (n = 18, 27.7%) ranked second and third, respectively, among reasons for not seeking physician care, indicating that health system factors relating to availability and accessibility predominantly resulted in unmet needs for this domain of care. Lack of financial resources also contributed to unmet needs for medicines (n = 6, 35.3%), ranking second, and was the main reason for unmet needs for investigations (n = 21, 87.5%). Personal factors ranked among the top three reasons for unmet needs for medicines and investigations. Personal responsibilities that deterred persons from obtaining anti-diabetic medication accounted for nearly 30% and considering care irrelevant accounted for 33%. Health system factors related to availability and accessibility predominantly influenced unmet needs in all three domains over personal factors.

Table 4 describes the observed associations between predisposing, enabling, and need factors and unmet needs for physician care, medicines, and investigations. For each domain of unmet needs, we compared those who reported unmet needs with those who did not, adjusting for covariates using logistic regression models.

**Table 4:** Binomial logistic regression analysis of predisposing, enabling and need factors associated with unmet needs for physician care, medicines, and investigations.

	Total	Unmet needs for physician visits				Unmet needs for medicines				Unmet needs for investigations			
		n	%	AOR(CI)	p-value	n	%	AOR(CI)	p-value	n	%	AOR(CI)	p-value
**Predisposing factors**													
Age													
> 60	167	23	13.8	1		6	3.6	1		6	3.6	1	
≤ 60	234	42	17.9	1.41(0.76–2.61)	0.279	11	4.7	1.38(0.43–4.43)	0.583	18	7.7	2.13(0.75–6.04)	0.157
Sex													
Male	169	32	18.9	1		10	5.9	1		13	7.7	1	
Female	232	33	14.2	0.50(0.27–0.92)	0.026*	7	3.0	0.53(0.17–1.67)	0.277	11	4.7	0.72(0.28–1.82)	0.490
Education													
≤ Grade 10	150	25	16.7	1		9	6.0	1		10	6.7	1	
> Grade 10	251	40	15.9	0.97(0.54–1.74)	0.927	8	3.2	0.51(0.18–1.44)	0.203	14	5.6	0.72(0.29–1.76)	0.469
Marital status													
No	65	12	18.5	1		4	6.2	1		3	4.6	1	
Yes	336	53	15.8	0.72(0.32–1.58)	0.406	13	3.9	0.67(0.17–2.64)	0.566	21	6.2	1.48(0.37–5.97)	0.584
**Enabling factors**													
Resident area													
Rural	365	63	17.3	1		16	4.4	1		22	6.0	1	
Urban	36	2	5.6	0.36(0.08–1.59)	0.177	1	2.8	0.94(0.11–3.10)	0.953	2	5.6	1.26(0.26–6.15)	0.775
Income level													
Low	257	51	19.8	1		13	5.1	1		18	7.0	1	
Not declared	51	6	11.8	0.49(0.19–1.26)	0.139	0	0	0	0.997	1	2.0	0.19(0.02–1.53)	0.119
High	93	8	8.6	0.37(0.16–0.83)	0.016*	4	4.3	1.03(0.30–3.56)	0.963	5	5.4	0.68(0.23–2.02)	0.494
Employment													
No	254	37	14.6	1		10	3.9	1		15	5.9	1	
Yes	147	28	19.0	1.22(0.66–2.27)	0.521	7	4.8	1.12(0.35–3.59)	0.844	9	6.1	0.83(0.32–2.21)	0.715
Regular provider													
None	39	1	2.6	1		3	7.7	1		1	2.6	1	
Yes, public	192	42	21.1	0.67(0.27–1.66)	0.391	12	6.2	0.52(0.12–2.31)	0.391	15	7.5	0.24(0.07–0.89)	0.032*
Yes, private	170	22	12.0	0.39(0.15–1.01)	0.053	2	1.2	0.11(0.02–0.77)	0.026*	8	4.3	0.18(0.05–0.68)	0.012*
**Need factors**													
Self-assessed health status													
Bad	220	44	20.0	1		13	5.9	1		12	15.4	1	
Good	181	21	11.6	0.57(0.31–1.04)	0.068	4	2.2	0.38(0.11–1.32)	0.130	12	3.7	0.35(0.12–1.00)	0.051
Co-morbidity													
Yes	83	18	21.7	1		7	8.4	1		14	4.4	1	
No	318	47	14.8	0.70(0.36–1.37)	0.299	10	3.1	0.36(0.12–1.12)	0.079	10	12.0	0.31(0.12–0.80)	0.016*

*p<0.05.

Physicians are at the centre of care provision for patients with diabetes mellitus. In the multivariable analysis, two factors were independently associated with lower unmet needs for physician care. These were being female (OR:0.50, CI: 0.27–0.92), or having a higher income (OR:0.37, CI: 0.16–0.83).

Reduced likelihood of having unmet needs for medicines was associated only with having a regular care provider in the private sector (OR:0.11, CI: 0.02–0.77).

Having a regular care provider, either in the government (OR:0.24, CI: 0.07–0.89) or the private sector (OR: 0.18, CI: 0.05–0.68) and being free from other chronic illnesses (OR:0.31, CI:0.12–0.80) were associated with lower unmet need for investigations.

## Discussion

While Sri Lanka has made substantial progress towards universal health care, there is limited understanding of the experiences of individuals with non-communicable diseases like diabetes mellitus [44]. To the best of our knowledge, this study is the first to examine the level and determinants of unmet healthcare needs of patients with diabetes mellitus type 2 in Sri Lanka, in terms of physician care, medicines, and investigations during the preceding year. The only similar data we can find are from the 2015 STEP survey. However, it differed in several important respects. While the STEP survey recruited individuals aged 18–69 years with self-reported diabetes mellitus, we included those aged 40–69 years with a confirmed diagnosis using medical records. The STEP survey determined the number not on medication by a question on current use of medicines whereas we asked about instances where respondents failed to obtain the medicines prescribed by their diabetes care provider. We additionally investigated unmet needs for physician care and investigations.

One-fifth of people with diabetes mellitus reported experiencing an unmet need within the preceding twelve months, which is similar to or less than that reported in high-income countries [31,45,46]. Notably, forgoing physician visits was the most common type of unmet need, particularly among patients receiving services from the government sector. Four out of the ten independent variables included in the models predicted one or more types of unmet needs. Among these, two variables, namely sex and income, were correlated with unmet needs for physician care. However, contrary to findings in other countries women experienced less unmet needs [34,37,47].

An examination of reasons for unmet need reveals issues amenable to action. The main barrier to accessing physician care and medicines was the inability to obtain care at times that were convenient, given other demands on patients’ time. This is something that could be addressed by changing the times that facilities operate, currently at specified times during weekdays. The opportunity cost is likely a significant concern for economically active men, those working in the informal sector, and individuals with low incomes. Even though health services are provided free in the public sector, poorer households still face additional costs such as travel costs to reach the hospital [6]. These factors collectively help explain the lower level of unmet needs among females, who are less likely to participate in the formal workforce, and those with higher incomes. Individuals with higher incomes are also more likely to be able to access and use private sector service providers, where they have flexibility to schedule visits at their convenience.

Only a small proportion of people reported an unmet need for medicines (4.2%), which is consistent with the government policy of providing free care at the point of delivery, including medicines. Though not directly comparable, the STEP survey reported that 30.5% of that self-reporting diabetes were not on medication. The reasons for the difference could be attributed to the variations between the two studies stated previously.

Manne-Goehler et al [48] analysed mostly STEPS data from 28 low- and middle- income countries (excluding Sri Lanka) and reported that 62% of individuals with diabetes aged 18 to 69 years experienced an unmet need for treatment. However, their definition of treatment for diabetes was a composite measure of those who had received lifestyle modification advice and/or anti-hyperglycaemic medication. Furthermore, the denominator for calculating unmet need included those undiagnosed at the time of the survey, whereas our study included only those with a verifiable diagnosis of diabetes mellitus.

Considering that our study asked about unmet needs for medicines without making a distinction between anti-hyperglycaemic medication and other medicines for controlling concomitant conditions, such as other cardiovascular risk factors, prescribed by the diabetes care provider, we also examined evidence for unmet needs for similar conditions. Marcus et al [49] adopts a similar methodology to Manne-Goehler et al [48] to assess the unmet need for treatment of hypercholesterolemia in 35 LMICs and reported that Sri Lanka is an outlier, with much better access to medicines than other Southeast Asia and Western Pacific countries.

The requirement to visit public sector clinics every month to obtain medicines, regardless of disease status, creates an important barrier. Due to the reasons mentioned above, patients may skip clinic visits, thus missing the opportunity to obtain freely dispensed medication in public sector clinics and given the cost of medicines in the private sector, some patients may forgo medicines until their next clinic visit. The consequences were seen in the finding that those with a regular private provider had less unmet needs for medicines, likely because they can obtain them at their convenience from private pharmacies.

The unmet need for investigations was also infrequent at only 6.0%. The affordability of tests can be an issue for patients accessing public sector health facilities. Although most investigations are provided free at government hospitals, HbA1c, essential for monitoring diabetes, is not yet widely provided in them and can be expensive in the private sector. However, the Ministry of Health has now recognized it as an essential laboratory investigation that all government hospitals must provide. Having a regular provider for diabetes care reduced the likelihood of having an unmet need for investigations, which is intuitive. The likelihood of unmet needs increased with the presence of another significant chronic disease, aligning with findings in the published literature on unmet needs [39]. However, our study failed to find evidence to support any association between unmet need for medicines or investigations with any socio-demographic characteristics of patients, perhaps due to the low number of individuals that experienced unmet needs. Although the impact of perceived health status on reported unmet needs has been documented in the literature [37,50], our study did not find any association between unmet need and perceived heath status.

Having a regular care provider, particularly in the private sector, is associated with fewer unmet needs. Similar findings have been reported in high-income countries [31,45], but this factor is now emerging as important in low- and middle-income countries with pluralistic and fragmented health systems. A regular care provider ensures continuity of care, which is crucial for the quality of services received by individuals with chronic diseases [45]. According to the published literature, having a regular care provider increases accessibility to health services [51], enhances satisfaction with care [52], reduces the frequency of emergency room visits [53] and hospitalizations [54], and has a positive impact on the management of chronic conditions such as diabetes mellitus [45].

In the Sri Lankan context, obtaining services from the private sector allows individuals to be seen by the same doctor, establishing a strong doctor-patient relationship, and ensuring continuity of care. On the other hand, service delivery in the public sector does not guarantee seeing the same doctor, even if the patient visits the same facility. The ongoing primary care reforms aim to address this issue by assigning a primary care doctor to each citizen [16]. This new primary healthcare delivery model has the potential to address the unmet needs of persons with diabetes mellitus in Sri Lanka by removing the availability and accessibility barriers identified in this study. Ensuring access to a primary care provider closer to home, within the primary care institutions, for individuals with diabetes mellitus type 2 would reduce unmet needs when coupled with access to the required anti-diabetic medicines and investigations in the same healthcare setting.

Although, a policy is in place to guide the new reforms [16], progress has been slow [15], further impeded by the COVID-19 pandemic and the current economic crisis of Sri Lanka. It is crucial for health planners to prioritize the new reforms within a constrained fiscal environment. It is justifiable to say that adults with other chronic illnesses may encounter similar unmet needs reported for the persons with diabetes mellitus in this study. A considerable proportion of the adult population suffering from chronic diseases, including diabetes mellitus, could benefit from service delivery reforms and experience fewer unmet needs, leading to better health outcomes.

### Limitations

This study has several limitations. All measures of unmet need were self-reported, which introduces the possibility of recall bias.

Our definition of a regular place of care may not fully capture the complex therapeutic itineraries followed by many patients, which often involve multiple healthcare providers. This limitation may affect our understanding of access to care and the assessment of unmet needs.

We were not able to take account of the presence of other co-morbidities and their potential impact on access to care. Individuals with additional conditions may have different experiences and knowledge regarding obtaining care. While we collected data on co-morbidities, the complex picture revealed by the data and the lack of information on duration or severity in our relatively small sample prevented us from fully considering this factor.

Perception of unmet needs may vary according to respondents’ lived experiences, their expectations of health systems, and of health itself, thus making its assessment a subjective evaluation, which may be independent of access to healthcare. The influence of biases on reported associations cannot be entirely controlled for in the study. Furthermore, the sample size and composition limited our ability to identify differences between certain categories of variables.

## Conclusion

Our study provides valuable insights into the unmet needs for diabetes mellitus type 2 in Sri Lanka. Given the predominantly publicly financed and delivered healthcare system, it is critical to understand why a sizeable proportion of patients with diabetes experience an unmet need for physician care, although to a lesser extent for medicines and investigations.

The findings underscore the importance of having a regular healthcare provider who can provide continuity of care. This aspect of care is crucial for managing diabetes mellitus and other non-communicable diseases, as well as promoting patient-centred models of care that support treatment adherence.

The socio-economic differences observed highlight that individuals with higher incomes are less affected by unmet needs. This emphasizes the need to address disparities in access to care and ensure that individuals from all socio-economic backgrounds have equitable access to needed health services.

Expanding public sector coverage alone is not enough to address the growing burden of diabetes mellitus and the challenges associated with interrupted care. It is essential to address gaps in care provision and improve the accessibility of necessary health services for individuals with diabetes mellitus.

## Supporting information

S1 Text Unmet needs questionnaire. (DOCX)
