# Supplementary material for: Predictors of unmet needs among people with diabetes mellitus type 2 in Gampaha district of Sri Lanka
Source: PLOS Glob Public Health. 2024 Oct 10;4(10):e0002462. doi: 10.1371/journal.pgph.0002462 (PMC11466436; doi:10.1371/journal.pgph.0002462)
Supplement: S1 Text — (DOCX) [file pgph.0002462.s001.docx]

# Unmet healthcare needs for diabetes mellitus type 2

1. In the last 12 months, how many times were you unable to meet your diabetes care provider even when you wanted to?

| None | 0 |
| --- | --- |
| Once | 1 |
| Twice | 2 |
| Three times | 3 |
| Four or more times | 4 |
| Can’t remember | 99 |

1. What was the main reason/s for not been able to see your diabetes care provider? Multiple responses allowed. Rank from 1 to many.

| Lack of funds to pay for care |  |
| --- | --- |
| Lack of transport to get to the provider |  |
| Lack of availability during feasible times |  |
| Had other responsibilities to attend to |  |
| Lack of a person to accompany |  |
| Felt not necessary |  |
| Did not trust the providers |  |
| Long waiting time |  |
| Long travel time to get to the provider |  |
| Other, please specify |  |

1. In the last 12 months, how many times were you unable to get medicine/insulin prescribed by your diabetes care provider?

| None | 0 |
| --- | --- |
| Once | 1 |
| Twice | 2 |
| Three times | 3 |
| Four or more times | 4 |
| Can’t remember | 99 |

1. What was the main reason/s for not been able to obtain the medicines/insulin prescribed by your diabetes care provider? Multiple responses allowed. Rank from 1 to many.

| Lack of funds to pay for medicines |  |
| --- | --- |
| Lack of transport to get to the provider |  |
| Had other responsibilities to attend to |  |
| Lack of availability during feasible times |  |
| Lack of a person to accompany |  |
| Felt not necessary |  |
| Did not trust the providers |  |
| Long waiting time |  |
| Long travel time to get to the provider |  |
| Did not know from where to obtain medicines |  |
| Other, please specify |  |

1. In the last 12 months, were you unable to get the investigations (blood tests such as fasting blood sugar and HbA1c etc) prescribed by your diabetes care provider for managing your diabetes?

| None | 0 |
| --- | --- |
| Once | 1 |
| Twice | 2 |
| Three times | 3 |
| Four or more times | 4 |
| Can’t remember | 99 |

1. What was the main reason/s for not been able to get these investigation/s done? Multiple responses allowed. Rank from 1 to many.

| Lack of funds to pay for investigations |  |
| --- | --- |
| Lack of transport to get to the provider |  |
| Had other responsibilities to attend to |  |
| Lack of availability during feasible times |  |
| Lack of a person to accompany |  |
| Felt not necessary |  |
| Did not trust the providers |  |
| Long waiting time |  |
| Long travel time to get to the provider |  |
| Did not know from where to get the investigations done |  |
| Other, please specify |  |
